# Sequential Carbide Precipitation During Tempering and Its Influence on Strength–Toughness Balance in 31CrMoNiNbV Secondary Hardening Martensitic Steel

**DOI:** 10.3390/ma19153204

**Published:** 2026-07-27

**Authors:** Fengping Zhao, Yanjie Mou, Jie Hu, Xiying Ma, Xiaofei Guo

**Affiliations:** 1 School of Materials Science and Engineering, Shanghai University, Shanghai 200444, China; 2 State Key Laboratory of Advanced Special Steel, Shanghai University, Shanghai 200444, China

**Keywords:** secondary hardening martensitic steel, tempering temperature, carbide precipitation, mechanical properties

## Abstract

The temperature-dependent carbide precipitation behavior and mechanical properties in 31CrMoNiNbV secondary hardening martensitic steel were investigated over a tempering temperature range of 200–650 °C. Sequential carbide precipitation behavior was characterized using scanning electron microscopy, electron backscatter diffraction and transmission electron microscopy, which was further correlated with tensile properties, hardness and low-temperature toughness. At the high tempering temperature of 580 °C, the investigated material achieved a well-balanced combination of tensile strength of 1633 MPa and total elongation of 15.1%, wherein tempering-induced M_2_C precipitation contributed to strength retention, while dislocation recovery and the formation of high-angle grain boundaries led to concurrent improvement in ductility and toughness. A predictive framework linking tempering temperature to carbides evolution and the resultant mechanical properties has been established for the investigated material, guiding the microstructural and heat treatment parameter design of ultra-high strength secondary hardening martensitic steels.

## 1. Introduction

Ultra-high strength steels (UHSSs) have drawn attention for transportation, aerospace and energy applications, where structural reliability and lightweight design are highly demanded [1,2,3]. Secondary hardening martensitic steels can be strengthened during tempering by various carbides including M_23_C_6_, M_2_C, M_7_C_3_ or MC [4,5,6], making them promising candidates for applications requiring excellent strength–toughness combinations. However, the types, precipitation sites, and volume fraction of these precipitates are highly sensitive to tempering parameters [7,8,9,10], making a thorough understanding of carbide precipitation behavior and its effects on mechanical properties crucial for optimizing the performance of secondary hardening steels.

Previous research work revealed that at low tempering temperatures (100–300 °C), precipitation occurs primarily involving ε-carbide and M_3_C formation while preserving the as-quenched martensitic structure [11,12]. As tempering temperature increases, enhanced diffusion kinetics enable progressive carbide evolution coupled with martensite recovery and dislocation rearrangement [13]. Lin et al. [14] observed rod-like M_3_C formation at lath boundaries in 45Cr5MoVNb steel at 550 °C, accompanied by martensitic lath coarsening and impact toughness deterioration. In Cr–Mo hot-work die steels, Mo_2_C and Cr_7_C_3_ were observed transforming to thermodynamically stable Mo_6_C and Cr_23_C_6_ in the temperature range of 550–600 °C [4,15]. Dong et al. [16,17,18] investigated a 25Cr1MoVNbTiN steel and revealed that rod-like M_2_C (enriched in Mo and V) precipitates led to strong material hardening at 400 °C and pronounced softening occurred above 600 °C. Hui et al. [19] observed the complete precipitation of supersaturated carbon from martensite when tempering above 500 °C in Cr–Mo–V high-strength steels. These fine and dispersed Mo/V carbides strengthen the material by pinning dislocations effectively and the material strength further increases with increased volume fraction of these carbides [20]. The secondary hardening effect becomes more pronounced as Mo content increases, as Mo also provides solid solution strengthening and tends to segregate at prior austenite grain boundaries (PAGBs) to enhance grain boundary cohesion force in addition to the precipitation-hardening effect [21].

Despite these advances, clear knowledge gaps remain regarding the correlation between carbide precipitation, microstructural evolution, and mechanical properties. The temperature-dependent thermodynamic stability of various carbides and their specific effects on mechanical performance lack investigation [22,23,24]. Most previous studies on Nb–V microalloyed steels and Cr–Mo–V steels have focused on conventional low-alloy systems with single microalloying treatments [16,25], whereas limited attention has been paid to high-Cr, high-Mo martensitic steels with combined Nb and V microalloying. The developed 31CrMoNiNbV steel contains 3.01 wt.% Cr and 2.00 wt.% Mo along with additional Nb and V elements. High Cr content facilitates carbides precipitation, whereas high Mo expands the thermal stability range of M_2_C. In addition, the Nb and V microalloying may effectively refine the microstructure and affect grain boundary characteristics [25]. The synergy among these alloying elements is therefore expected to yield a carbide precipitation sequence and temperature-dependent stability window distinct from those reported for conventional Cr–Mo steels [23,24]. M_2_C carbides usually form at high tempering temperatures, as the diffusion of Mo, V, and Cr elements needs to be enhanced [17]. As temperature further increases, M_2_C could also dissolve and transform into other types of carbides. The temperature thresholds triggering specific carbide transformations and their corresponding effects on strength–toughness balance need to be identified for the optimization of heat treatment parameters.

This study investigates the temperature-controlled sequential carbide precipitation behavior in a 31CrMoNiNbV secondary hardening martensitic steel across a tempering temperature range of 200–650 °C. Carbide evolution and the critical temperature thresholds that govern specific transformation sequences are investigated using scanning electron microscopy (SEM) and transmission electron microscopy (TEM). The impacts of carbide evolution on mechanical properties and fracture behaviors are discussed accordingly, which provide references for strength–toughness optimization in secondary hardening martensitic steels.

## 2. Materials and Methods

The investigated material is a medium-carbon secondary hardening steel (designated as 31CrMoNiNbV) with Cr, Ni, and Mo as primary alloying elements, supplemented with Nb and V to promote grain refinement and precipitation hardening. The chemical composition is listed in Table 1. The heat treatment was performed through three steps: (i) normalizing at 1060 °C for 40 min in a Nabertherm LT 24/12 muffle furnace (Lilienthal, Germany) followed by air cooling; (ii) austenitization at 1040 °C for 60 min followed by oil-quenching; (iii) tempering at different temperatures respectively (200 °C, 300 °C, 400 °C, 500 °C, 520 °C, 540 °C, 560 °C, 580 °C, 600 °C, 620 °C, 650 °C) using a Nabertherm LT 15/14 muffle furnace for 120 min, followed by water-cooling to room temperature with a cooling rate estimated between 200 and 300 °C/s.

Microstructure characterization was conducted using SEM (ZEISS Gemini460, Oberkochen, Germany) with energy dispersive spectrometer (EDS) on polished surface etched with a 4% (vol.%) nitric acid alcoholic solution for 5 s. The crystal orientation and grain boundary features were characterized under electron backscatter diffraction (EBSD, Oxford Instruments, Abingdon, UK) mode. Specimens were mechanically polished and finally polished with a 0.02 µm colloidal silica suspension for approximately 30 min to minimize residual surface stresses. EBSD maps were acquired with a step size of 0.1 µm. High-resolution transmission electron microscopy (TEM, Thermo Fisher Talos F200X, Waltham, MA, USA) with EDS (XFlash Detector 5030) was utilized to characterize the lath morphology and precipitated carbides. The specimens were prepared using a Struers Tenupol-5 electrolytic double-jet apparatus (Ballerup, Denmark) with a 10 vol.% perchloric acid–ethanol electrolyte at −30 °C and further thinned using a Gatan 691 precision ion polishing system (Pleasanton, CA, USA). In addition to characterization, the types and volume fractions of carbides in equilibrium condition were also predicted by thermodynamic calculations using Thermo-Calc software with TCFE11 database. Dislocation density after different tempering treatments was quantified by X-ray diffraction (XRD, Rigaku Smartlab, Tokyo, Japan) using Cu–Kα radiation at 40 kV over a 2*θ* range of 20–110° at 1°/min scanning speed. The (110)α, (200)α, (211)α, and (220)α diffraction peaks were selected for classical Williamson–Hall analysis [26].

Mechanical properties were evaluated by uniaxial tensile testing, Charpy V-Notch impact test, and Rockwell hardness measurement. Smooth cylindrical tensile specimens (diameter: Φ5 mm; gauge length: 35 mm; specimen axis parallel to the rolling direction) were tested at a strain rate of 5 × 10^−4^ s^−1^ using a universal testing machine (Instron 5985, Norwood, MA, USA). Charpy V-notch impact testing was performed at −40 °C on specimens (10 mm × 10 mm × 55 mm with a notch radius of 0.25 mm and depth of 2 mm) using an impact testing machine (Instron MPX750, Norwood, MA, USA). Rockwell hardness (HRC) was measured using a diamond indenter under 150 kgf load with 2 s dwell time (Wilson RH2150, Buehler, Lake Bluff, IL, USA). All tests were repeated three times to ensure statistical reliability.

## 3. Results

### 3.1. Microstructural Evolution During Tempering Treatment

**Figure 1:**
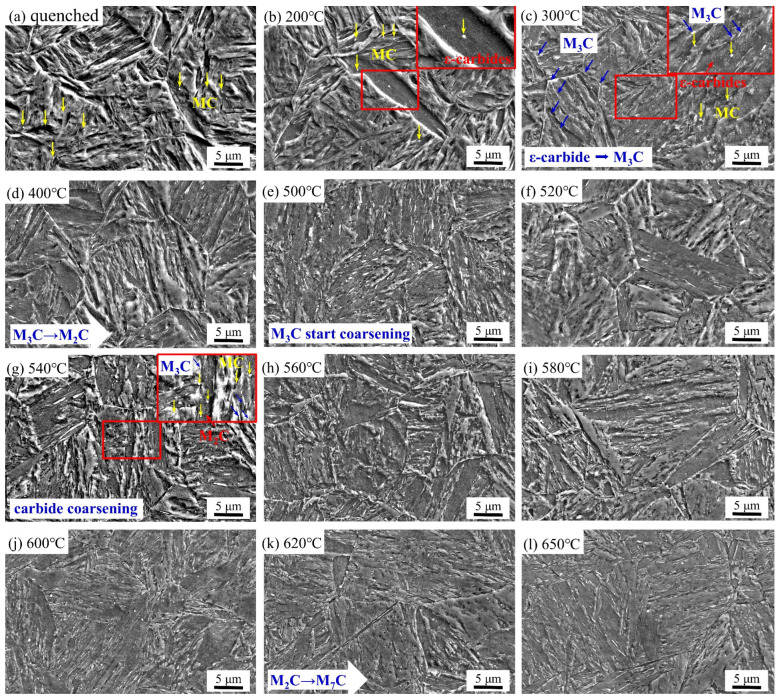
Microstructure and carbide evolution after quenching and subsequent tempering at 200–650 °C for 120 min: (**a**) as-quenched; tempered at (**b**) 200 °C; (**c**) 300 °C; (**d**) 400 °C; (**e**) 500 °C; (**f**) 520 °C; (**g**) 540 °C; (**h**) 560 °C; (**i**) 580 °C; (**j**) 600 °C; (**k**) 620 °C; (**l**) 650 °C.

**Figure 2:**
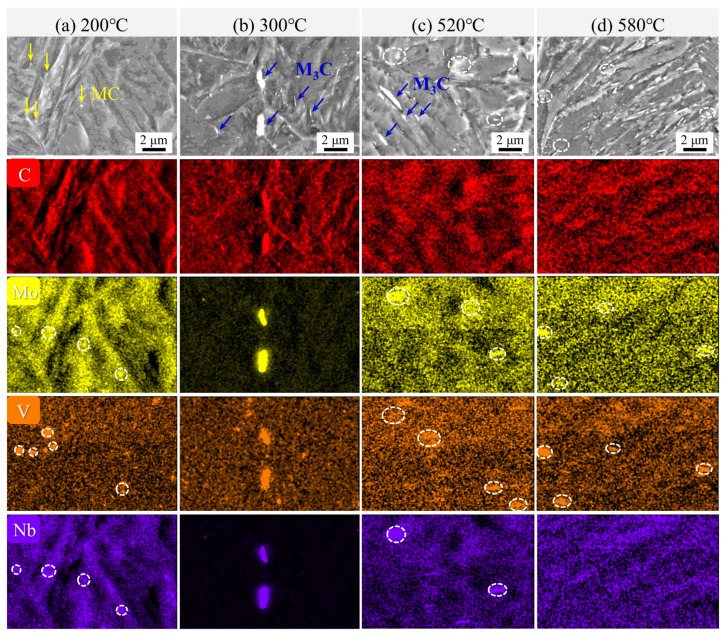
SEM images and EDS elemental maps (C, Mo, V, Nb) of the microstructure after tempering between 200 and 580 °C for 120 min: (**a**) 200 °C; (**b**) 300 °C; (**c**) 520 °C; (**d**) 580 °C. (The write dashed circles indicate carbide precipitation).

**Figure 3:**
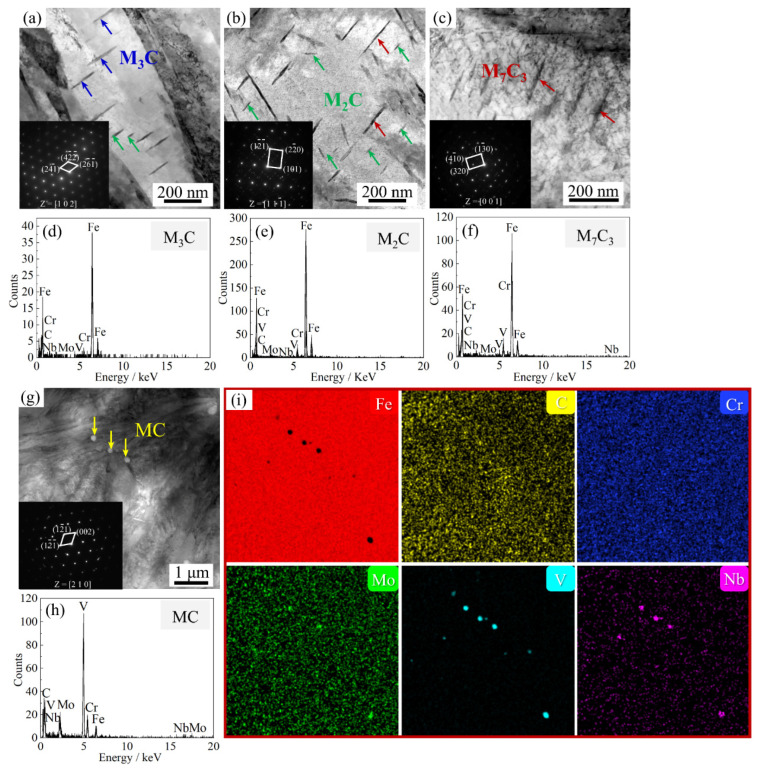
TEM micrographs and corresponding SAED patterns of the 31CrMoNiNbV steel after tempering at: (**a**) 500 °C; (**b**) 580 °C; (**c**) 620 °C; (**d**–**f**) corresponding EDS spectra of M_3_C, M_2_C and M_7_C_3_ carbides; (**g**–**i**) bright-field image, SAED pattern, and EDS elemental mapping of MC precipitates after tempering at 580 °C.

Figure 1 shows the microstructural and carbide morphologies in 31CrMoNiNbV steel in the as-quenched condition and after tempering between 200 and 650 °C for 120 min. As shown in Figure 1a, the as-quenched specimen exhibits a predominantly martensitic matrix containing spherical carbides. These carbides remain stable throughout the entire tempering range and are identified as V/Nb/Mo-enriched MC-type carbides by EDS mapping (Figure 2a) and TEM analysis (Figure 3g). Tempering at 200 °C induces finely dispersed ε-carbides within martensite laths (Figure 1b). Above 300 °C, needle-shaped M_3_C-type carbides precipitate along lath boundaries (Figure 1c), identified as Fe-rich M_3_C containing Mo, V, and Nb, as confirmed by TEM bright-field imaging and EDS analysis in Figure 3a and Figure 3d respectively. With increasing tempering temperature, M_3_C carbides progressively coarsened to micrometer-scale dimensions (Figure 1d).

**Figure 4:**
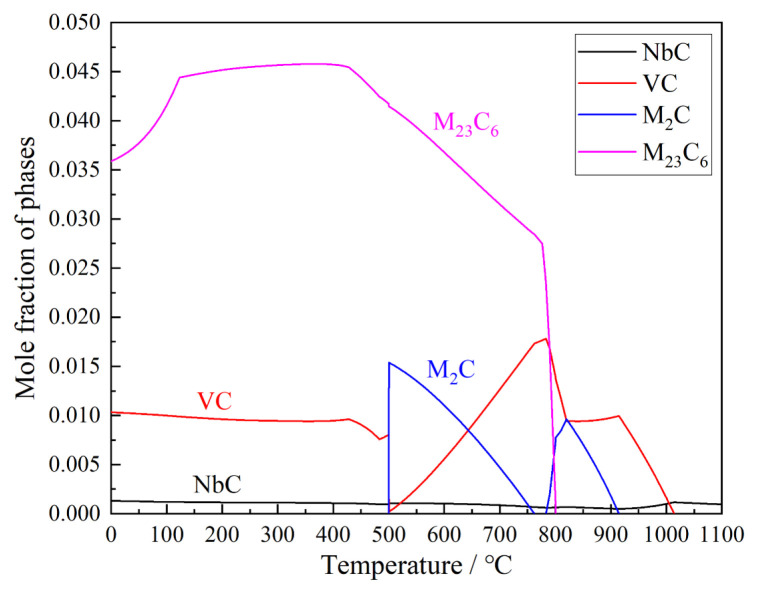
Equilibrium phase diagram of carbides at different temperatures calculated by the Thermo-Calc software (TCFE11 database).

At ~400 °C, sequential carbide transformation (ε-carbide → M_3_C → M_2_C) initiates within martensite laths. Short rod-like nanoscale M_2_C carbides enriched in Fe with minor Mo, Cr, and V are clearly observed at ~500 °C [27], as confirmed by EDS analysis, TEM micrographs, and selected area electron diffraction (SAED) patterns in Figure 3b,e. Between 500 and 580 °C, extensive complex carbide precipitation occurs throughout the matrix (white dashed circles, Figure 2). After tempering at 580 °C, M_2_C gradually transforms into elongated, linear M_7_C_3_ precipitates (Figure 3c), with EDS analysis in Figure 3f confirming that M_7_C_3_ is in Fe-based phase with Cr and Mo composition. Concurrently, large blocky M_23_C_6_ carbides form along grain and lath boundaries (Figure 1e–l). Above 600 °C, M_3_C gradually dissolves while extensive M_2_C → M_7_C_3_ transformation occurs (Figure 1j–l). The size of M_7_C_3_ carbides is 0.1–2.5 μm in length. MC carbides remain stable throughout the entire tempering range, distributing primarily within laths [16], which are consistent with Thermo-Calc predictions (Figure 4). MC carbides are identified as V/Nb/Mo-enriched composite precipitates, and the inflection observed in the corresponding Thermo-Calc curve may signify competitive precipitation among these elements.

Table 2 summarizes the sequential carbide precipitation across different tempering temperature ranges. At low temperatures, ε-carbides, M_3_C and MC-type carbides dominate. From 500 °C onward, M_2_C progressively replaces M_3_C, accompanied by concurrent M_23_C_6_ precipitation. Above 580 °C, M_3_C diminishes as substantial M_2_C transforms to M_7_C_3_, while MC carbides exist at all tempering temperatures.

**Figure 5:**
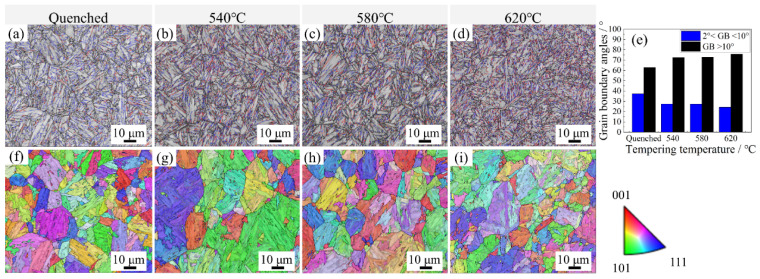
EBSD analysis of the as-quenched and tempered (540–620 °C for 120 min) microstructures: image quality maps and inverse pole figure maps of (**a**,**f**) as-quenched; (**b**,**g**) 540 °C; (**c**,**h**) 580 °C and (**d**,**i**) 620 °C; (**e**) fractions of LAGBs (blue) and HAGBs (black) at different tempering temperatures.

Figure 5 presents EBSD analysis of the 31CrMoNiNbV steel in the as-quenched state and after tempering at 540 °C, 580 °C and 620 °C. Grain boundaries are classified by misorientation angle: low-angle grain boundaries (LAGBs, <10°, blue lines), and high-angle grain boundaries (HAGBs, ≥10°, black lines), as shown in Figure 5a–d. The HAGB fraction increases progressively with tempering temperature, from 62.8% in the as-quenched state to 72.6% at 540 °C, 72.9% at 580 °C, and 75.8% at 620 °C respectively (Figure 5e). Inverse pole figure (IPF) maps in Figure 5f–i reveal random crystal orientation and the average prior austenite grain size of ~15 μm.

### 3.2. Dislocation Densities

**Figure 6:**
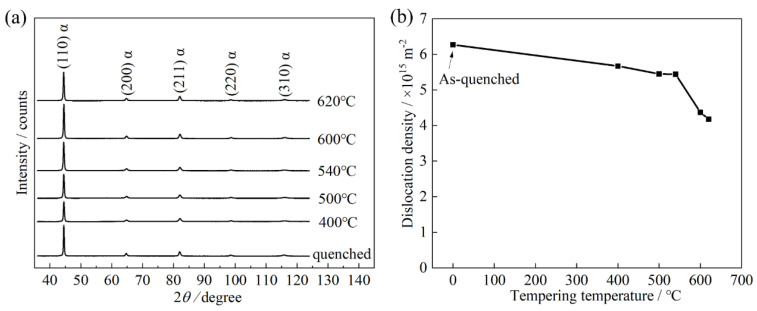
XRD spectra (**a**) and the calculated dislocation densities (**b**) after quenching and tempering at 400–620 °C for 120 min.

Figure 6a presents the XRD spectra of the 31CrMoNiNbV steel after tempering at various temperatures, confirming a predominantly martensitic microstructure. Dislocation density was calculated using the classical Williamson–Hall (W–H) method [26], with the W–H equation expressed as:

(1) $$\frac{\Delta 2\theta \cos\theta }{\lambda }=\frac{0.9}{D}+2\varepsilon \frac{\sin\theta }{\lambda }$$

where *λ* is the X-ray wavelength (0.154 nm), D is the apparent grain size, and *ε* is the lattice strain. The dislocation density *ρ* is calculated as [28]:

(2) $$\rho =\frac{2\sqrt{3}\varepsilon }{\mathrm{Db}}$$

where *ρ* is the dislocation density and b is the magnitude of the Burgers vector (0.248 nm for steel). The linear fit (Δ2*θ*cos*θ*/*λ* versus 2sin*θ*/*λ*) has an intercept of 0.9/D on the Δ2*θ*cos*θ*/*λ* axis. By combining with Equation (2), the dislocation density of the experimental steel can be determined. Figure 6b shows the dislocation density at different tempering temperatures, with a value of 6.27 × 10^15^ m^−2^ in the as-quenched condition. When the tempering temperature rises to 540 °C, the dislocation density decreases from 6.27 × 10^15^ m^−2^ to 5.44 × 10^15^ m^−2^, with a reduction of approximately 13.2%. Upon further heating to 600 °C, the dislocation density drops to 4.37 × 10^15^ m^−2^, corresponding to a reduction of around 19.7%. It can be seen that the dislocation density exhibits a larger reduction in the temperature range of 540–600 °C.

### 3.3. Tensile Properties

**Figure 7:**
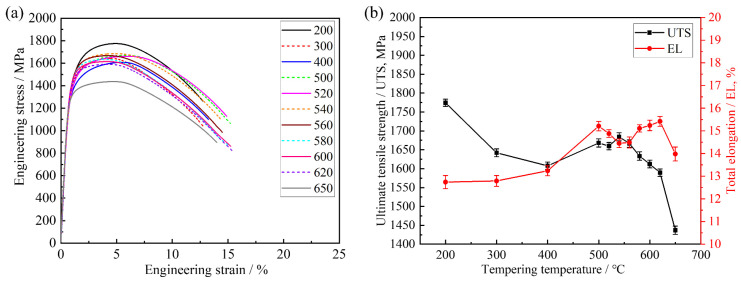
Tensile properties of the 31CrMoNiNbV steel tempered at different temperatures: (**a**) engineering stress–strain curves; (**b**) ultimate tensile strength and total elongation as a function of tempering temperature.

Tempering temperature significantly influences microstructural evolution and carbide precipitation behavior, resulting in large variations in the mechanical properties of the 31CrMoNiNbV steel. Figure 7 presents the tensile properties of the specimens after tempering at different temperatures. Figure 7a shows the engineering stress–strain curves with the tensile properties summarized in Figure 7b. After tempering at 200 °C, the steel achieved an ultimate tensile strength (UTS) of 1774 MPa and 12.7% total elongation. After tempering at 300 °C, the UTS decreased steeply to 1642 MPa. At 400 °C, the UTS decreases further by 166 MPa while elongation improves slightly to 13.2%. A relatively high elongation (15.2%) is obtained after tempering at 500 °C, while peak UTS (1684 MPa) is reached at 540 °C with 14.5% elongation. The ultimate tensile strength reaches its peak at 540 °C owing to the high co-precipitation amount of M_2_C and M_3_C carbides at this temperature, which yields greater precipitation strengthening effect compared with the carbide precipitation condition at 500 °C. Beyond 540 °C, strength declines progressively while elongation improves from 14.5% to 15.4%. At 650 °C, the UTS dropped notably to 1437 MPa with an elongation of 14.0%.

**Figure 8:**
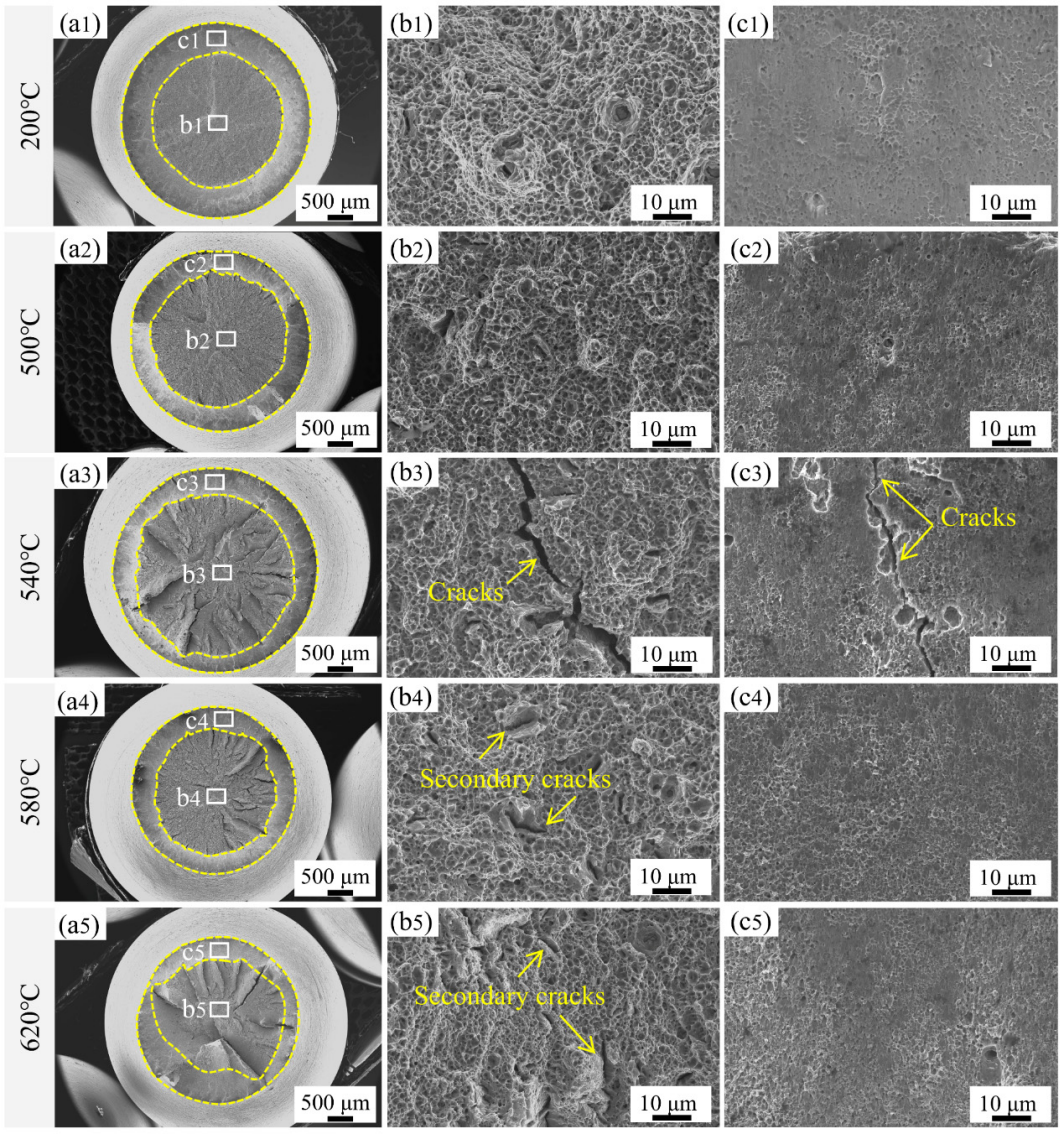
SEM characterization of fracture surfaces from tensile specimens: (**a1**–**c1**) 200 °C; (**a2**–**c2**) 500 °C; (**a3**–**c3**) 540 °C; (**a4**–**c4**) 580 °C; (**a5**–**c5**) 620 °C.

Figure 8 presents fracture surfaces of tensile specimens. All specimens exhibited a typical ductile dimple fracture with some observable microcracks. Shear lip regions (between yellow dashed lines) at the specimen periphery account for area fractions of 53.3% (200 °C), 43.4% (500 °C), 37.3% (540 °C), 43.4% (580 °C), and 44.7% (620 °C) respectively. At 200 °C, the fracture surface consists of a shear lip region (Figure 8(c1)) surrounding a central fibrous region (Figure 8(b1)) containing shallow dimples (Figure 8(a1)). At 540 °C, numerous cracks appear in the central fibrous region (Figure 8(a3)) and extend into the shear lip region (Figure 8(c3)). At 580 °C, short micro-cracks in the central region are observed alongside radial macro-cracks (Figure 8(b4)). At 620 °C, delamination cracks emerge, with the central fibrous region showing abundant dimples and secondary cracks (Figure 8(b5)).

### 3.4. Hardness and Low-Temperature Impact Toughness

**Figure 9:**
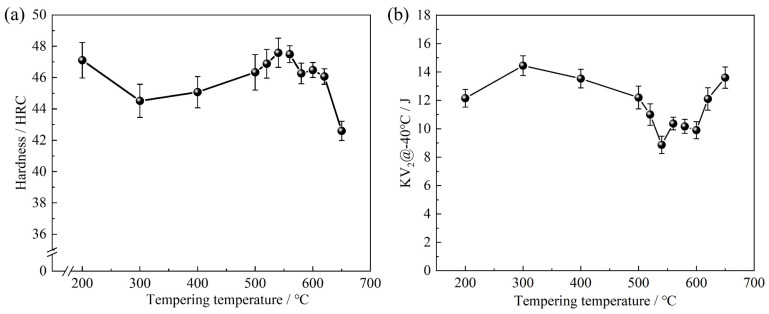
Rockwell hardness (**a**) and −40 °C Charpy impact toughness (**b**) of 31CrMoNiNbV steel after tempering at 200–650 °C for 120 min.

Figure 9 shows the variation in Rockwell hardness and −40 °C impact toughness of the 31CrMoNiNbV steel with tempering temperature. As shown in Figure 9a, the hardness decreases from 47.1 HRC (200 °C) to 44.5 HRC (300 °C) after 120 min tempering. Between 300 °C and 540 °C, the sequential carbide transformation (ε-carbide → M_3_C → M_2_C) within martensitic laths progressively raises the hardness to 47.6 HRC at 540 °C, which is attributed to M_2_C precipitation strengthening [29]. Hardness remains stable from 540 °C to 620 °C, then declines to 42.6 HRC at 650 °C due to reduced dislocation density and carbide coarsening [30]. Figure 9b displays −40 °C impact toughness of V-notched specimens tempered at different temperatures. It rises from 12.2 J to 14.4 J when the tempering temperature increases from 200 °C to 300 °C, and this initial toughness improvement is mainly associated with the dissolution of brittle ε-carbides and the decrease in supersaturated solute atoms in martensite, which effectively alleviates the intrinsic brittleness of the matrix, and subsequently declines to a minimum of 8.9 J at 540 °C. Between 540 °C and 600 °C, it stays at a plateau of ~10 J, which further increases to 13.6 J at 650 °C.

**Figure 10:**
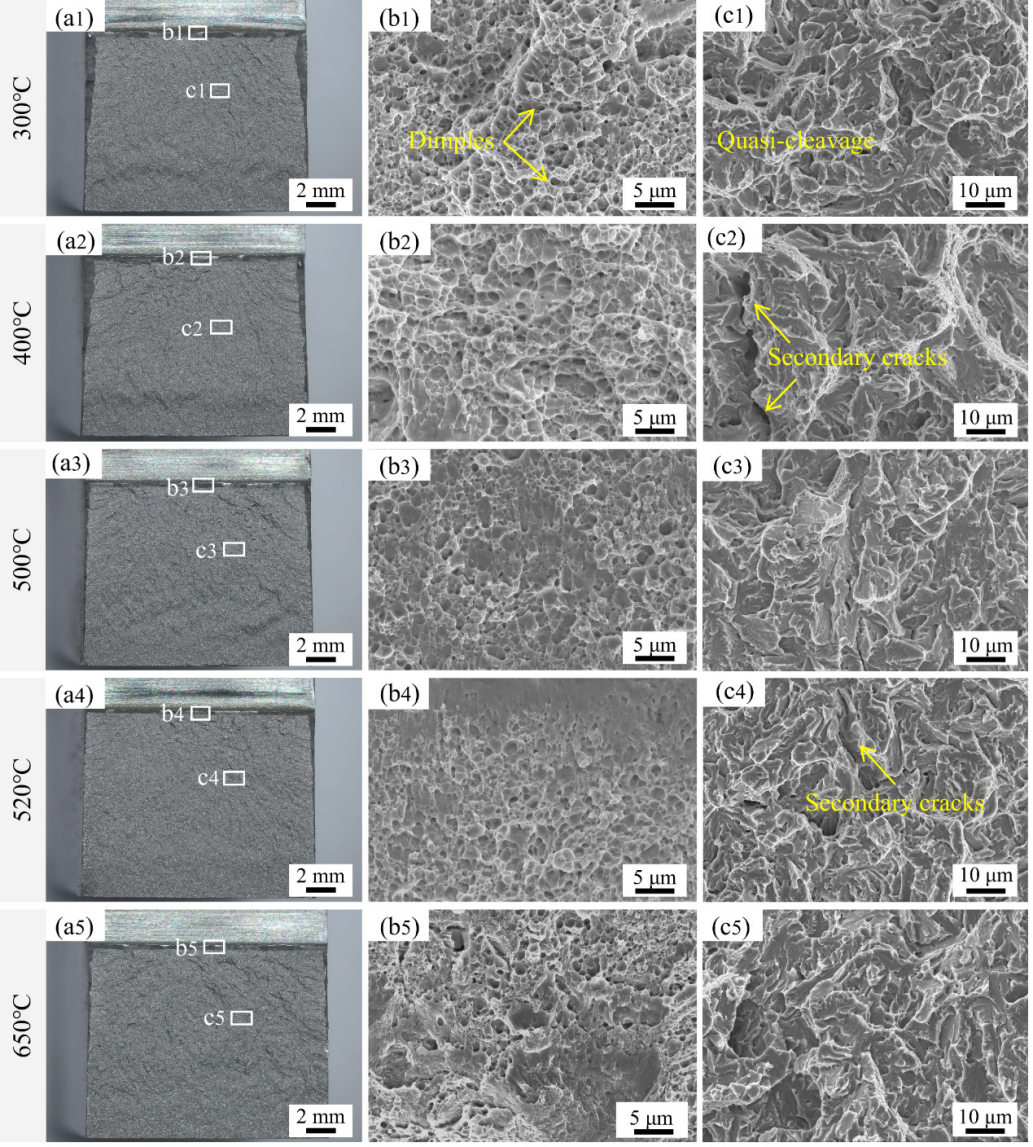
Fracture surfaces of Charpy impact specimens after tempering at different temperatures: (**a1**–**c1**) 300 °C; (**a2**–**c2**) 400 °C; (**a3**–**c3**) 500 °C; (**a4**–**c4**) 520 °C; (**a5**–**c5**) 650 °C.

Figure 10 shows the fracture surfaces of charpy impact specimens tested at −40 °C. Macroscopic fracture surfaces (Figure 10(a1–a5)) display a flat, gray fibrous appearance with distinct radial crack patterns. Microscopic analysis near the notch root (Figure 10(b1–b5)) and in the crack propagation regions (Figure 10(c1–c5)) reveals transitional fracture behavior with increasing tempering temperature. Between 300 and 520 °C, small shallow dimples dominate near the notch root. At 650 °C, localized tearing ridges appear, indicating a transition from quasi-cleavage to ductile fracture and improved impact toughness. The crack propagation regions exhibit similar quasi-cleavage fracture features across all tempering temperatures.

## 4. Discussion

### 4.1. Carbide Evolution During Tempering Process

Tempering temperature significantly influences the type and precipitation sites of carbides. SEM and TEM characterizations confirm MC carbides as a stable phase throughout the entire tempering range. Previous studies suggest that fine MC carbides precipitate within the ferrite matrix, satisfying the Baker–Nutting orientation relationship with ferrite and contributing to strength, toughness, and tempering resistance [31]. These carbides, formed primarily from V/Nb/M_O_, enhance strength and high-temperature toughness while inhibiting the coarsening of transitional carbides [32].

At lower tempering temperatures (300–400 °C), orthorhombic M_3_C carbides preferentially precipitate along martensite lath boundaries as a transient phase, governed by rapid carbon diffusion and limited substitutional element mobility. M_3_C accommodates considerable substitutional elements; for instance, Cr progressively replaces Fe with increasing temperature [10]. Although M_3_C is hard and brittle, potentially inducing brittleness and poor machinability, these detrimental effects can be mitigated through optimized alloying composition [33]. At 150–250 °C, supersaturated carbon precipitates from martensite as transitional ε-carbide, which gradually transforms into M_3_C. Above 400 °C, M_3_C begins to coarsen (Figure 1d), and subsequently decomposes to M_2_C and M_7_C_3_ [16].

**Figure 11:**
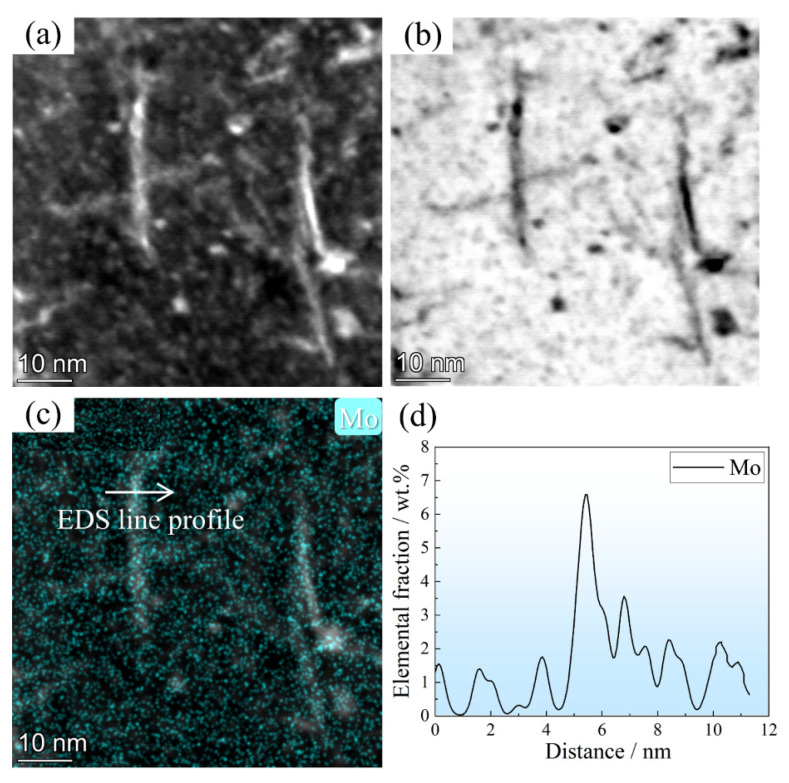
TEM characterization of M_2_C precipitates in 580 °C tempered specimen: (**a**,**b**) dark-field and bright-field images showing nanoscale M_2_C precipitates; (**c**) EDS mapping of Mo; (**d**) EDS line profile of Mo along the line marked in (**c**).

M_2_C carbides have a hexagonal close-packed (hcp) crystal structure and usually precipitate above 400 °C [34]. Initially, Guinier–Preston (G–P) zones of Mo and C clustering along {100} planes form coherent interfaces, contributing to secondary hardening [35]. M_2_C carbides nucleate through two mechanisms: independent nucleation at dislocation sites, forming fine needle-like precipitates, or nucleating in situ on pre-existing M_3_C carbides to form coarser needle-shaped structures [34]. M_2_C formation is mainly governed by Mo diffusion, which is significantly enhanced above ~500 °C (Figure 11). At higher temperatures, coarse cementite plates decompose due to in situ precipitation of fine Mo_2_C particles.

M_7_C_3_ carbides form via in situ nucleation through dissolution and transformation of M_2_C at ~580 °C, precipitating primarily within laths and coarsening rapidly [36,37]. In the investigated steel, M_7_C_3_ (orthorhombic structure) is detected above 580 °C (Figure 3c) and attributed to preferential Cr diffusion into M_2_C, as reported in other tempered martensitic steels (4.5Cr, 2W, 0.25V, 0.1C) [4]. Janovec et al. revealed that carbide evolution in Cr-containing steels depends strongly on Cr content [7]. In medium-to-high Cr steels, M_23_C_6_ is more stable than M_7_C_3_, whereas in Cr–Mo–V steels with ~1 wt.% Cr, M_7_C_3_ exhibits high thermal stability [4,7,33]. With 3.01 wt.% Cr in the investigated steel, M_23_C_6_ could be thermodynamically more stable than M_7_C_3_. M_23_C_6_ carbides have a cubic structure and precipitate along grain and subgrain boundaries at ~500 °C (Figure 1e) [38]. Their tendency to coarsen and form continuous boundary networks is detrimental to toughness and impact resistance. M_23_C_6_ consists primarily of Cr and Fe, with partial Fe substitution by Cr, potentially influencing carbide stability [16,33,39].

**Figure 12:**
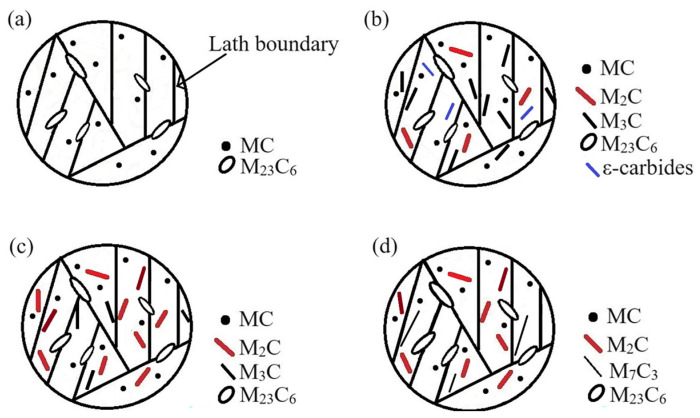
Schematic illustration of carbide evolution in the 31CrMoNiNbV steel across different tempering temperature ranges: (**a**) as-quenched; tempered at (**b**) 200–400 °C; (**c**) 400–580 °C; (**d**) 580–650 °C.

Figure 12 illustrates the carbide precipitation sequences observed at different tempering temperature ranges. Carbide transformation proceeds through two fundamental mechanisms [16,40]: (i) in situ separation, where new carbides nucleate at original carbide/matrix interfaces and grow at the expense of the precursor phase; (ii) separate nucleation, where new carbides nucleate at defect sites (primarily dislocations) while original carbides dissolve. Current work reveals that M_2_C, M_23_C_6_ and M_7_C_3_ mainly form via in situ separation, while M_3_C and MC mainly follow separate nucleation. ε-carbide is a metastable transition phase that forms in the range of 150–200 °C, where carbon segregates at lattice defects in supersaturated martensite, undergoes ordering, and precipitates along coherent interfaces. Above 400 °C, ε-carbide dissolves and transforms into cementite (M_3_C) [41].

### 4.2. Influence of Carbide Evolution on Mechanical Properties

**Figure 13:**
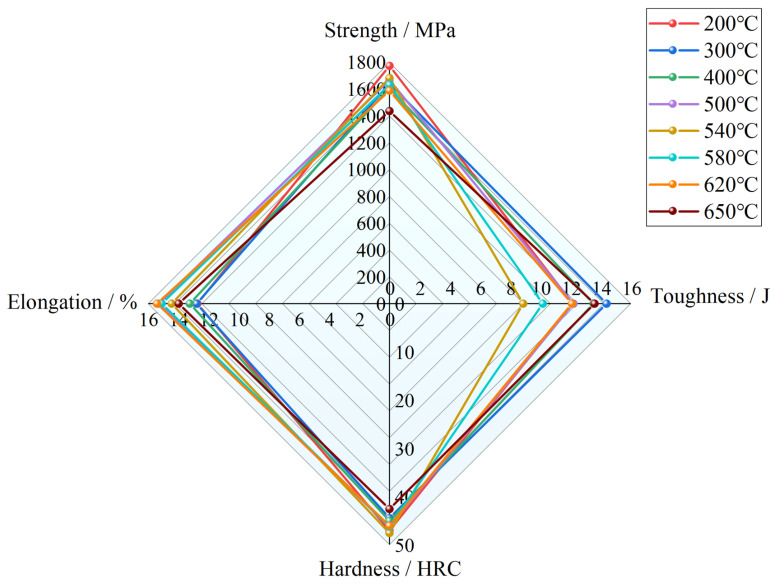
Radar chart comparing mechanical properties of the 31CrMoNiNbV steel across different tempering temperatures.

Following oil-quenching and tempering at 200 °C for 120 min, the steel achieves a UTS of 1774 MPa with 12.7% elongation, strengthened by high dislocation density and fine ε-carbide precipitation. Between 300 °C and 400 °C, elongated M_3_C carbides nucleate along martensite lath boundaries, inducing substantial toughness degradation [12], as shown in Figure 9b. Between 400 °C and 520 °C, ε-carbides dissociate and M_2_C forms [29], which nucleates predominantly at M_3_C/matrix interfaces [31] (Figure 11). Above 500 °C, both M_3_C and M_23_C_6_ coarsen along martensite packet boundaries, lath boundaries, and prior austenite grain boundaries. This preferential boundary precipitation weakens the carbide/matrix interfacial cohesion [18], causing reductions in both impact toughness and elongation (Figure 13). This progressive carbide transformation leads to the peak hardness (47.6 HRC) and peak tensile strength (1684 MPa) (Figure 9a); however, it results in a toughness minimum (8.9 J) at 540 °C, attributed to extensive boundary carbide coarsening, which reduces both impact toughness and tensile elongation (Figure 13) [42].

**Figure 14:**
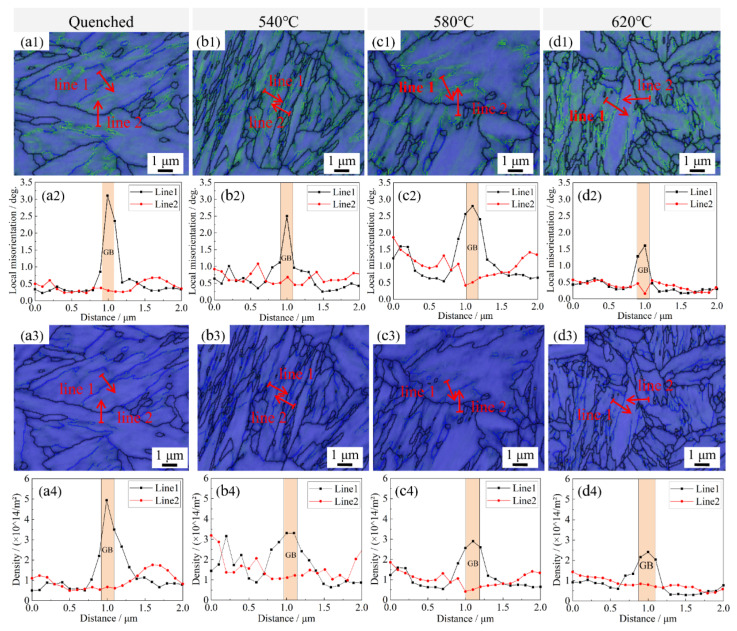
EBSD analysis of microstructure after quenching and tempering at 540–620 °C for 120 min: (**a1**–**d1**) KAM maps showing elevated local misorientation around LAGBs; (**a2**–**d2**) local misorientation profiles across grain boundaries taken from regions marked in (**a1**–**d1**); (**a3**–**d3**) GND maps showing elevated dislocation density around LAGBs; (**a4**–**d4**) dislocation density profiles across grain boundaries taken from regions marked in (**a3**–**d3**).

Impact toughness improves between 540 °C and 580 °C (Figure 9b) due to substantial dislocation recovery and annihilation, which partially mitigates the detrimental effects of boundary carbides. At 580–620 °C, both impact toughness (10.2 J to 12.1 J) and total elongation (15.1% to 15.4%) increase as tempering temperature increases (Figure 13). Figure 14 shows that regions adjacent to LAGBs exhibit elevated local misorientation and dislocation density in both the as-quenched and tempered conditions, decreasing progressively with increasing tempering temperature. Furthermore, the HAGB fraction increases with tempering temperature (Figure 5e). Previous work suggests that an increased HAGB fraction promotes crack deflection at cleavage boundaries, thereby enhancing toughness [43].

Lv et al. [44] demonstrated that martensitic laths are the fundamental structural units governing strength in Cr–Ni–Mo–V/Nb high-strength steel, which possesses a multi-alloy composition system similar to that of the present 31CrMoNiNbV steel. The increasing tempering temperature reduces dislocation density and softens the martensitic matrix [45]. However, material strength depends not only on lath width and dislocation density, but also on solid solution and precipitation strengthening. Below 500 °C, the limited diffusivity of carbide-forming elements contrasts with facile carbon diffusion, resulting in predominant M_3_C precipitation. Between 500 °C and 600 °C, enhanced elements diffusion enables M_2_C precipitation, inducing secondary hardening and strength recovery. In this temperature range, the highest product of strength and ductility is achieved at 580 °C with a UTS of 1633 MPa, a total elongation of 15.1% and a low-temperature impact toughness of 10.2 J (Figure 13). At higher tempering temperatures, both strength and hardness decline due to reduced dislocation density and carbide coarsening [14]. The 650 °C tempered specimen has UTS of 1437 Mpa and hardness of 42.6 HRC, while the impact toughness recovers to 13.6 J.

## 5. Conclusions

This study elucidates the temperature-controlled carbide precipitation behavior and the correlation with mechanical properties in 31CrMoNiNbV secondary hardening martensitic steel. The main findings are summarized as follows:

(1) Temperature-dependent carbide evolution in 31CrMoNiNbV steel proceeds through distinct transformation sequences. MC carbides exhibit good thermal stability across 200–650 °C. Sequential ε-carbide–M_3_C–M_2_C transformations initiate at 200 °C and complete at ~540 °C, inducing secondary hardening (peak hardness: 47.6 HRC at 540 °C) with degraded toughness (8.9 J). Above 300 °C, M_3_C precipitation along lath boundaries deteriorates both strength and toughness. In the range of 500–540 °C, coarsening of M_3_C and M_23_C_6_ at boundaries raises hardness; however, it deteriorates impact toughness. Above 580 °C, M_3_C decomposition and M_2_C to M_7_C_3_ transformations occur concurrently with substantial dislocation recovery and grain/subgrain boundary restructuring.
(2) In the high tempering temperature range, the specimen tempered at 580 °C achieved a relatively good strength–toughness balance (UTS of 1633 MPa, total elongation of 15.1% and −40 °C impact toughness of 10.2 J), attributed to substantial dislocation recovery, progressive M_3_C decomposition and M_2_C formation eliminating detrimental grain boundary carbides, and increased HAGB fraction promoting crack deflection. Consequently, high strength is preserved while tempering embrittlement is partially mitigated.
(3) The established temperature-dependent carbide evolution map provides guidelines for designing heat treatment protocols to achieve tailored strength–toughness combinations through controlled carbide precipitation in ultra high-strength secondary hardening martensitic steels.

## Figures and Tables

**Table 1:** Chemical composition of the investigated 31CrMoNiNbV steel (wt.%).

Material	C	Si	Mn	P	S	Cr	Ni	Mo	Nb	V	Fe
31CrMoNiNbV	0.31	0.041	0.19	0.0082	0.0008	3.01	0.92	2.00	0.085	0.46	Bal.

**Table 2:** Carbide evolution across different tempering temperatures.

Tempering Temperature	200 °C	300 °C	400 °C	500 °C	580 °C	620 °C
	MC	MC	MC	MC	MC	MC
Carbide types	ε-carbides	ε-carbides→M_3_C	M_3_C→M_2_C	M_3_C→M_2_C		
		M_3_C	M_3_C	M_3_C→M_2_C	M_3_C→M_2_C	
	M_23_C_6_	M_23_C_6_	M_23_C_6_	M_23_C_6_	M_23_C_6_	M_23_C_6_
				M_2_C	M_2_C→M_7_C_3_	M_2_C→M_7_C_3_

## Data Availability

The original contributions presented in this study are included in the article. Further inquiries can be directed to the corresponding author.

## References

[1] Niu M.C., Yin L.C., Yang K., Luan J.H., Wang W., Jiao Z.B. (2021). Synergistic alloying effects on nanoscale precipitation and mechanical properties of ultrahigh-strength steels strengthened by Ni_3_Ti, Mo-enriched, and Cr-rich co-precipitates. Acta Mater..

[2] Gao Y.H., Liu S.Z., Hu X.B., Ren Q.Q., Li Y., Dravid V.P., Wang C.X. (2019). A novel low cost 2000 MPa grade ultra-high strength steel with balanced strength and toughness. Mater. Sci. Eng. A.

[3] Zhao Y.J., Ren X.P., Yang W.C., Zang Y. (2013). Design of a low-alloy high-strength and high-toughness martensitic steel. Int. J. Miner. Metall. Mater..

[4] Asadabad M.A., Kheirandish S., Novinrooz A.J. (2010). Microstructural and mechanical behavior of 4.5Cr-2W-0.25V-0.1C steel. Mater. Sci. Eng. A.

[5] Rao M.P., Sarma V.S., Sankaran S. (2013). Development of high strength and ductile ultra fine grained dual phase steel with nano sized carbide precipitates in a V-Nb microalloyed steel. Mater. Sci. Eng. A.

[6] Li J., Zhan D., Jiang Z., Zhang H., Yang Y., Zhang Y. (2023). Progress on improving strength-toughness of ultra-high strength martensitic steels for aerospace applications: A review. J. Mater. Res. Technol..

[7] Janovec J., Vyrostkova A., Svoboda M. (1994). Influence of tempering temperature on stability of carbide phases in 2.6Cr-0.7Mo-0.3V steel with various carbon content. Metall. Mater. Trans. A.

[8] Zhang C., Wang Q., Ren J., Li R., Wang M., Zhang F., Yan Z. (2012). Effect of microstructure on the strength of 25CrMo48V martensitic steel tempered at different temperature and time. Mater. Des..

[9] Han P., Liu Z., Xie Z., Wang H., Jin Y., Wang X., Shang C. (2023). Influence of band microstructure on carbide precipitation behavior and toughness of 1 GPa-grade ultra-heavy gauge low-alloy steel. Int. J. Miner. Metall. Mater..

[10] Tao X., Gu J., Han L. (2014). Characterization of precipitates in X12CrMoWVNbN10-1-1 steel during heat treatment. J. Nucl. Mater..

[11] Claesson E., Magnusson H., Hedström P. (2023). Scanning precession electron diffraction study of carbide precipitation sequence in low alloy martensitic Cr-Mo-V tool steel. Mater. Charact..

[12] Hou Z., Babu R.P., Hedström P., Odqvist J. (2019). Early stages of cementite precipitation during tempering of 1C-1Cr martensitic steel. J. Mater. Sci..

[13] Claesson E., Magnusson H., Kohlbrecher J., Thuvander M., Hedström P. (2022). Evolution of iron carbides during tempering of low-alloy tool steel studied with polarized small angle neutron scattering, electron microscopy and atom probe. Mater. Charact..

[14] Lin G.B., Zhao P., Wei A. (2019). Effect of tempering temperature on microstructure and properties of medium carbon alloy steel 4Cr5MoSiV1Nb. Iron Steel.

[15] Yuan Y., Wang W., Zhou M., Shi R., Wang Z., Zhang Y., Xie J. (2025). Microstructure stability and softening resistance of a novel Cr-Mo-V hot work die steel. High Temp. Mater. Process..

[16] Dong J., Zhou X., Liu Y., Li C., Liu C., Guo Q. (2017). Carbide precipitation in Nb-V-Ti microalloyed ultra-high strength steel during tempering. Mater. Sci. Eng. A.

[17] Song H.Y., Hui W.J., Fang B.Y., Zhao Z.B., Zhang Y.J., Zhao X.L. (2024). Enhanced mechanical properties of a V+Nb-microalloyed medium-carbon steel by controlled forging. Mater. Sci. Eng. A.

[18] Kwon H. (1991). Comparison of Secondary Hardening Embrittlement in Tungsten and Molybdenum Steels. Metall. Trans. A.

[19] Hui W.J., Dong H., Weng Y.Q., Wang M.Q., Chen S.L., Shi J. (2002). Effect of heat treatment parameters on mechanical properties of high strength Cr-Mo-V steel. Acta Metall. Sin..

[20] Hui W.J., Dong H., Wang M.Q., Chen S.L., Weng Y.Q. (2002). Influence of quenching temperature on mechanical properties of low alloy high strength Cr-Mo-V steel. Heat Treat. Met..

[21] Hui W.J., Dong H., Weng Y.Q., Shi J., Zhang X.Z. (2004). Effect of molybdenum on delayed fracture behavior of high strength steel. Acta Metall. Sin..

[22] Xie Z.J., Shang C.J., Wang X.L., Wang X.M., Han G., Misra R.D.K. (2020). Recent progress in third-generation low alloy steels developed under M^3^ microstructure control. Int. J. Miner. Metall. Mater..

[23] Wang R., Li F.H., Wu Z.H., Kang Y., Fan J., Yu Z., Yan Z., Du S., Eckert J. (2024). Precipitation and transformation of carbides during tempering of 7Cr14 martensitic stainless steel. Steel Res. Int..

[24] Xie Y.X., Cheng X.N., Wei J.B., Luo R. (2022). Characterization of carbide precipitation during tempering for quenched dievar steel. Materials.

[25] Huang S., Li C.R., Li Z.Y., Zeng Z.Y., Zhai Y.Q., Wang J., Liu Z.L., Zhuang C.L. (2021). Quantitative analysis of microstructure and mechanical properties of Nb–V microalloyed high-strength seismic reinforcement with different Nb additions. High Temp. Mater. Process..

[26] Williamson G.K., Hall W.H. (1953). X-ray line broadening from filed aluminium and wolfram. Acta Metall..

[27] Mondiere A., Déneux V., Binot N., Delagnes D. (2018). Controlling the MC and M_2_C carbide precipitation in ferrium^®^ M54^®^ steel to achieve optimum ultimate tensile strength/fracture toughness balance. Mater. Charact..

[28] Williamson G.K., Smallman R.E. (1954). The use of fourier analysis in the interpretation of X-ray line broadening from cold-worked iron and molybdenum. Acta Crystallogr..

[29] Tao X.G., Han L.Z., Gu J.F. (2014). Effect of tempering on microstructure evolution and mechanical properties of X12CrMoWVNbN10-1-1 steel. Mater. Sci. Eng. A.

[30] Yoo C.H., Lee H.M., Chan J.W., Morris J.W. (1996). M_2_C precipitates in isothermal tempering of high Co-Ni secondary hardening steel. Metall. Mater. Trans. A.

[31] Zhang C.Y., Wang Q.F., Kong J.L., Xie G.Z., Wang M.Z., Zhang F.C. (2013). Effect of martensite morphology on impact toughness of ultra-high strength 25CrMo48V steel seamless tube quenched at different temperatures. J. Iron Steel Res. Int..

[32] Xie Z.J., Ma X.P., Shang C.J., Wang X.M., Subramanian S.V. (2015). Nano-sized precipitation and properties of a low carbon niobium micro-alloyed bainitic steel. Mater. Sci. Eng. A.

[33] Janovec J., Svoboda M., Výrostková A., Kroupa A. (2005). Time-temperature-precipitation diagrams of carbide evolution in low alloy steels. Mater. Sci. Eng. A.

[34] Zhao Z.Y. (2002). Studing status on the secondary hardening phenomenon in ultra-high strength steels. J. Aeronaut. Mater..

[35] Zhou H.Y., Sun X.R., Tong Z., Cheng G., Xu B.B., Xiao X., Wang Q., Ran M.R., Ding H., Zheng W.Y. (2024). Effects of tempering temperature on the precipitation behaviors of nanoparticles and their influences on the susceptibility to hydrogen embrittlement of a Cr-Mo-V steel. Int. J. Hydrogen Energy.

[36] Zhong J.X., Zheng X.H., Xu Z.R., Bai D.Z. (1989). Carbides in steel 4335V. Acta Metall. Sin..

[37] Wang M.Q., Dong H., Wang Q., Li J.X., Zhao L. (2003). Carbides in secondary hardening steel 25Cr3Mo3NiNb. J. Iron Steel Res..

[38] Liu J., Zhao F.P., Shi W., Dong H., Guo X.F. (2025). Enhanced hydrogen embrittlement resistance in a vanadium-alloyed 42CrNiMoV steel for high-strength wind turbine bolts. Acta Metall. Sin. (Engl. Lett.).

[39] Novinrooz A.J., Moniri S., Asadabad M.A., Hojabri A. (2012). The study of nano-sized carbide particles formed in Fe-Cr-W-V alloy. J. Mater. Eng. Perform..

[40] Wang M.M., Wei C.Y., Guo G.S., Li D.D., Liu S., Zhang H.Q. (2023). Research progress on strengthening, toughening and fatigue properties of ultra-high-strength martensitic-based steel. Trans. Mater. Heat Treat..

[41] Tsai M.C., Yang J.R. (2003). Microstructural degeneration of simulated heat-affected zone in 2.25Cr-1Mo steel during high-temperature exposure. Mater. Sci. Eng. A.

[42] Ayer R., Machmeier P. (1998). On the characteristics of M_2_C carbides in the peak hardening regime of AerMet 100 steel. Metall. Mater. Trans. A.

[43] Moura L.B., de Abreu H.F.G., Araújo W.S., Franco J.F.B., Sampaio M.C., Maurício F.E.R. (2018). Embrittlement and aging at 475 °C in an experimental superferritic stainless steel with high molybdenum content. Corros. Sci..

[44] Lv Z.W., Fan J., Wang R., Yu Z.Q., Kang Y., Hu Y., Tuo L.F., Eckert J., Yan Z.J. (2025). Microstructural evolution during tempering process and mechanical properties of Cr–Ni–Mo–V/Nb high strength steel. J. Iron Steel Res. Int..

[45] Deng B., Chen P., Wang G.D. (2021). Effect of tempering temperature on microstructure and properties of secondary hardening martensitic stainless steel. Heat Treat. Met..

